# The Pool of Siloam

**Published:** 1871-05

**Authors:** 


### The Pool of Siloam.

The miraculous efficacy of the Pool of Siloam, as recorded in the Scriptures is familiar to us all, but its modern condition appears as fraught with danger, as was its ancient with the power of healing. Speaking of a fatal case of enteric fever, the surgeon of H. M. S., *Endymion*, Dr. Alex. Fisher, says:

" I attribute the origin of this case to the use of the water at Jerusalem, and consider ourselves fortunate in having escaped with only one case of enteric fever among the seventy-two persons visiting it. Without the walls of Jerusalem the water appears to be very good, but inside it is received into vast tanks and reservoirs beneath the Harem area, and elsewhere. These, from what I saw in the excavations recently executed by the Palestine Exploration Society, are entirely without protection from receiving a large proportion of the sewage of the city, in some cases without even the slightest filtration through earth or other obstacle. At the fouutain of Siloam and Pool of Siloam, the water distinctly tasted like soap-suds, brought down by the water from the baths, etc., close to the Temple enclosure.

